# BAF57/SMARCE1 Interacting with Splicing Factor SRSF1 Regulates Mechanical Stress-Induced Alternative Splicing of Cyclin D1

**DOI:** 10.3390/genes12020306

**Published:** 2021-02-21

**Authors:** Jianguo Feng, Xichao Xu, Xin Fan, Qian Yi, Liling Tang

**Affiliations:** 1 Key Laboratory of Biorheological Science and Technology, Ministry of Education, College of Bioengineering, Chongqing University, Chongqing 401120, China; fengjianguo@swmu.edu.cn (J.F.); 20161901022@cqu.edu.cn (X.X.); 2 Department of Anesthesiology, The Affiliated Hospital of Southwest Medical University, Luzhou 646000, China; 3 Department of Anatomy, School of Basic Medical Sciences, Southern Medical University, Guangzhou 510515, China; 4 Department of General Surgery, Xinqiao Hospital, Army Medical University, Chongqing 401120, China; fanxin.5@163.com; 5 Department of Physiology, College of Preclinical Medicine, Southwest Medical University, Luzhou 646000, China; yiqian2010@yeah.net

**Keywords:** alternative splicing, BAF57/SMARCE1, *cyclin D1*, mechanical strain

## Abstract

**Background:** Cyclin D1 regulates cyclin-dependent protein kinase activity of the cell cycle, and *cyclin D1* alternative splicing generates a *cyclin D1b* isoform, acting as a mediator of aberrant cellular proliferation. As alternative splicing processes are sensitive to mechanical stimuli, whether the alternative splicing of *cyclin D1* is regulated by mechanical stress and what kinds of factors may act as the regulator of mechano-induced alternative splicing remain unknown. **Methods:** The alternative splicing of *Cyclin D1* was examined using reverse transcription polymerase chain reaction (RT-PCR) in osteoblast cell lines and keratinocyte cells loaded by a cyclic stretch. The expression of splicing factors and switching defective/sucrose non-fermenting (SWI/SNF) complex subunits were detected in stretched cells using real-time quantitative PCR (RT-qPCR). The protein interaction was tested by co-immunoprecipitation assay (Co-IP). **Results:** *Cyclin D1* expression decreased with its splice variant upregulated in stretched cells. Serine/arginine-rich splicing factor 1 (SRSF1) and SWI/SNF complex subunit Brahma-related gene-1-associated factor 57 (BAF57), also named SWI/SNF-related matrix-associated actin-dependent regulator of chromatin subfamily E member 1 (SMARCE1), could respond to mechanical stimuli. Overexpression and knockdown experiments indicated the BAF57/SMARCE1 is probably a critical factor regulating the alternative splicing of cyclin D1. Co-IP showed an interaction between BAF57/SMARCE1 and SRSF1, implying a possible underlying mechanism of the regulator role of BAF57/SMARCE1 in the splicing process of cyclin D1. **Conclusions:** The splicing factor SRSF1 and BAF57/SMARCE1 are possibly responsible for the mechanical stress-induced alternative splicing of cyclin D1.

## 1. Introduction

Gene alternative splicing is a physiologic process that enormously enriches the proteome, which plays an important role in organ development and function disorder. Alternative splicing contains four basic modules, including alternative 5′ splice-site choice, alternative 3′ splice-site choice, cassette-exon inclusion or skipping, and intron retention. It involves the use of one or more of four basic modules to create protein diversity [1]. Splicing factors SR proteins and hnRNPs could bind to splicing enhancers or splicing silencers in exons or flanking introns to influence the alternative splicing process [2]. Except for intrinsic factors, extracellular stimulus also regulates gene alternative splicing, such as mechanical stimuli.

Mechanical stimuli regulate the process of gene alternative splicing, generating gene variants which are crucial for physiological activities and pathological changes, such as inflammatory injury [3], muscle hypertrophy [4] and bone formation [5]. Mechanical stimuli, including stretch and fluid shear stress, modulate the alternative splicing of vascular endothelial growth factor-A (*VEGF-A*) [6], transcriptional factor *FosB* [7], *CD44* [8] etc. Roosa and colleagues [5] evaluated the extent of alternative splicing in a bone subjected to mechanical loading using Affymetrix exon arrays and found that the gene alternative splicing pattern in stress sensitive cells could be changed by mechanical stimuli. Our previous study also provided that insulin-like growth factor-1 (*IGF-1*) gene alternative splicing can be regulated by mechanical stress in osteoblast cells [9]. *IGF-1* is alternatively spliced to produce IGF-1 EC (also named MGF) mRNA variant after stress loaded. While MGF retains a very low level in normal physiological or culture condition, the expression of MGF dramatically increases in response to mechanical stimuli. Although, the interaction between splicing factors and splicing regulatory elements is crucial for the alternative splicing process, the research on splicing factors is insufficient for elucidating the molecular mechanisms whereby the gene alternative splicing is regulated by mechanical stress.

In recent years, the roles of the switching defective/sucrose non-fermenting (SWI/SNF) complex in alternative splicing process have attracted increasing attention. In mammalian cells, there are more than 10 subunits which constitute the SWI/SNF complex, including BAF53a (ACTL6A), BAF57 (SMARCE1), BAF60a (SMARCD1), BAF60b (SMARCD2), BAF60C (SMARCD3), BAF155 (SMARCC1), BAF170 (SMARCC2), Brg1 (SMARCA4), Brm (SMARCA2), SNF5ini (SMARCB1) etc., in which Brg1 or Brm exerts itself as the catalytic subunit [10,11]. The SWI/SNF complex was capable of regulating cell-cycle progression, DNA replication, development, differentiation, elongation, signaling, splicing, DNA-damage repair [12,13]. Underhill’s research first showed the association between the SWI/SNF complex and the spliceosome, indicating that four SWI/SNF complex subunits, including Brg1, Brm, BAF155 and SNF5/INI1, splicing factor SF3a120 and spliceosome associated protein SAP 130 were found to colocalize in the N-CoR-1 complex [14]. Batsche and colleagues [15] demonstrated that Brm, the core subunit of the SWI/SNF complex, was found to regulate the alternative splicing of *cyclin D1* and *CD44*, and co-immunoprecipitation assay showed the interaction of Brm with U5 SnRNAs and Sam68. Tyagi [16] further found that Brm is associated with nascent pre-mRNPs, and influences the levels of alternatively processed mRNAs through the microarrays experiments. This accumulating evidence indicates that SWI/SNF not only acts at the transcriptional level to regulate the amount of mRNAs synthesized from a given promoter, but also exerts itself at the post-transcriptional level to modulate the type of alternative transcript produced.

However, whether other subunits of SWI/SNF influence the alternative splicing process is still unknown. More recently, the SWI/SNF complex was identified as a mechanoregulated inhibitor of YAP and TAZ [17]. Therefore, in this study, we screen the subunits of the SWI/SNF complex’s response to mechanical stress, and investigate the functions of the subunits on regulating mechanotransduction-mediated alternative splicing.

## 2. Material and Methods

### 2.1. Cell Culture and Cell Stretch

The mouse osteoblast Mc3t3-E1 and human keratinocyte HaCaT cells were grown in Ham’s F12 medium or Roswell Park Memorial Institute (RPMI) 1640 medium supplemented with 10% fetal bovine serum at 37 °C in 5% CO_2_. The two cell lines were provided by Stem Cell Bank, Chinese Academy of Sciences. The osteoblast or skin cells were seeded on the surface of a silicone membrane, and the cyclic-loading experiments as we previously described were performed [18]. Briefly, the cells were cultured overnight, and stretched for 3 and 6 h, at a strain magnitude of 15% with a frequency of 30 cycles/min. Cells cultured on the silicone membranes with no cyclic-loading stress served as the control.

### 2.2. RT-PCR Analysis

Loaded cells were harvested for total RNA isolation by Trizol regent according to the manufacturer’s instructions. cDNA was synthesized from total RNA using PrimeScript^TM^ RT reagent kit with gDNA Eraser (Takara, Kusatsu, Japan) at 42 °C for 2 min, 37 °C for 30min, 85 °C for 5 s and 4 °C for 5min. The mRNA levels of SWI/SNF complex subunits were measured by quantitative real-time PCR using SYBR Green Dye (Takara, Japan), and the relative gene expression was calculated through the 2^−ΔΔCT^ method. The real-time quantitative PCR was conducted on a Bio-Rad CFX96 system (Bio-Rad, Hercules, CA, USA). β-actin served as the internal reference gene. The primers used for RT-qPCR analysis are shown in Table 1. 

The mRNA levels of *VEGF*, *CD44*, *cyclin D1* and spliced isoforms were detected by reverse transcription semi-quantitative PCR. The PCR reactions were performed at 95 °C for 5 min, followed by 40 cycles of 95 °C for 30 s, 54 °C for 30 s and 72 °C for 1 min, using the DNA polymerase purchased from Tiangen Biotech CO. LTD. (Beijing, China), with the specific primers which are also shown in Table 1. The PCR was conducted on a thermal cycler MyCycler (Bio-Rad, CA, USA). The PCR product was analyzed by 1.5% agarose gel electrophoresis. 

### 2.3. Transfection of Plasmidand siRNA

pcDNA3.1-BAF57 used in this study was prepared in our lab as previously reported [11,19]. BAF57/SMARCE1-specific siRNA was synthesized by GenePharma (Shanghai, China), and the sequence is 5’ AAGGAGAACCGUACAUGAGCA 3’ [11]. Plasmids were transiently transfected in HaCaT cells by Effectene Transfection Reagent (Qiagen, Hilden, Germany) and siRNAs were transfected in HaCaT cells using RNAiMAX Reagent (Life Technologies, Carlsbad, CA, USA) according to the manufacturer’s instructions. Cells were collected for subsequent examination at 48 h after transfection.

### 2.4. Western Blot

Cells were lysed by iced radioimmunoprecipitation assay (RIPA) buffer (50mM Tris pH 7.4, 150mM NaCl, 1% NP-40, 0.5% sodium deoxycholate, 1mM ethylenediamine tetraacetic acid (EDTA), 0.1% SDS) supplemented with 1 mM phenylmethylsulfonyl fluoride (PMSF). Protein concentration was measured by bicinchoninic acid (BCA) assay (Beyotime, Shanghai, China). Protein samples were subjected to SDS-PAGE electrophoresis and then transferred to polyvinylidene fluoride (PVDF) membrane. After being blocked with 5% skim milk in Tris-buffered saline plus Tween 20 (TBST) for 1h, the membranes were incubated with primary antibodies at 4 ℃ overnight, and then incubated with corresponding radish peroxidase-conjugated secondary antibodies (Proteintech, Wuhan, China) for 1 h at room temperature. Western blot bands were detected via West Pico Super Signal chemiluminescent substrate (Pierce, Rockford, IL, USA) using ChemiDoc XRS Imaging System (BioRad, Hercules, CA, USA). SRSF1 and β-actin antibodies were purchased from Santa Cruz. Variants were detected with anti-VEGF (Abcam, No.ab1316), anti-CD44 (ABclonal, No. A1351), and anti-Cyclin D1(CST, No. 2926P) antibodies, respectively. For relative quantification, the integrated optical density (IOD) was estimated using ImageJ (NIH). Relative protein expression level was calculated as IOD Experimental/IOD Control.

### 2.5. Immunoprecipitations, IP

Cells were seeded into 10-cm diameter dishes, and harvested until 70–80% confluent, then processed with a Dounce homogenizer in iced NP40 lysis buffer (50 mM Tris pH 7.4, 150 mM NaCl, 0.5% NP40, 1 mM EDTA) with 1 mM PMSF. After homogenization, the cell samples were placed on ice for 30 minutes followed by centrifugation at 14,000 rpm at 4 °C for 30 min. The protein extract was incubated with antibodies overnight, then protein A/G plus agarose beads (Santa Cruz, CA, USA) 40 μl slurry was added to each sample and incubated at 4 °C on an end-to-end rotator for 4 h. After the incubation, beads were triple-washed by lysis buffer and resuspended in 1× SDS-PAGE sample buffer (Beyotime, Shanghai, China). Samples were boiled at 95℃ for 10 min before loaded to 10% SDS-PAGE for Western blot analysis.

### 2.6. RNA Immunoprecipitation (RIP) Assay

The RIP assay was carried out according to our previous study [19]. Briefly, 1 × 10^7^ cells were harvested and lysed in nuclear isolation buffer (1.28 M sucrose, 40 mM Tris-HCl pH 7.5, 20 mM MgCl_2_, 4% Triton X-100) to obtain the nuclear pellet. Then the nuclear pellet was mechanically sheared by a dounce homogenizer, which was suspended in RIP buffer (150 mM KCl, 25 mM Tris pH 7.4, 5 mM EDTA, 0.5 mM Dithiothreitol (DTT), 0.5% NP40, 100 U/mL RNAase inhibitor, protease inhibitor cocktail). SMARCE1 antibody was added to the supernatant after nuclear membrane and debris were discarded by centrifugation, and incubated at 4°C overnight. Subsequently, 40 μL Protein-A/G beads were added with gentle rotation at 4°C for 2 h. The beads were then resuspended in 1 mL TRIzol reagent after triple RIP buffer washing. Total RNA was extracted according to and transcribed into cDNA. PCR was performed to detect cyclin D1 with the primers (forward: GAGGAGCAGAAGTGCGAAGA, and reverse: TGGAGGGTGGGTTGGAAA). 

### 2.7. Statistics

All data were presented as the mean ± SD. The Statistical Package for the Social Sciences 19.0 (SPSS) was used for the statistical analyses. Differences between two groups were determined by Student’s t-test. The Benjamini and Hochberg (BH) method was applied for multiple testing correction. All experiments were repeated at least three times. *p* values less than 0.05 were considered statistically significant differences.

## 3. Results

### 3.1. The SWI/SNF Complex Responded to Mechanical Strain

**Figure 1:**
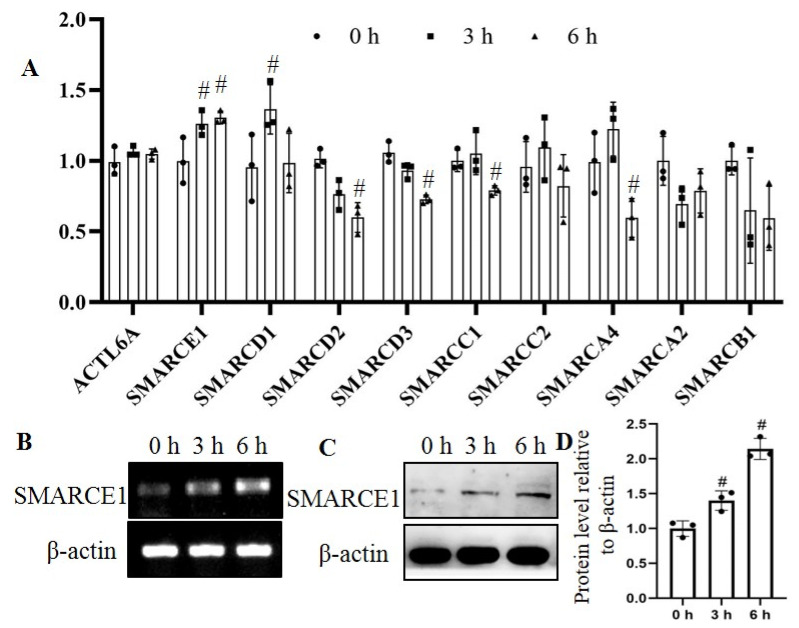
The mechanical strain affected the expression of SWI/SNF complex subunits. (**A**). Mc3t3-E1 cells were cyclic-stressed for 3 and 6 h, and RT-qPCR was used to examine the levels of SWI/SNF complex subunits. The expression of SMARCE1 was tested by reverse transcription semi-quantitative PCR (**B**) and Western blotting (**C**) in respond to mechanical stimulation at 3 and 6 h. (**D**) The protein levels of SMARCE1 showed in C were quantified by Image J software. The data represent three sets of independent experiments (*n* = 3) and were shown as means ±SD. #*p* < 0.05 vs. the 0 h group.

RT-qPCR was used to examine the expression alteration of SWI/SNF complex subunits in osteoblast MC3t3-E1, including *ACTL6A, SMARCE1, SMARCD1, SMARCD2, SMARCD3, SMARCC1, SMARCC2 SMARCA4, SMARCA2* and *SMARCB1*. As shown in Figure 1A, *SMARCE1* was gradually upregulated at 3 h (26.0%) and 6 h (30.5%) after mechanical strain, while *SMARCD1* was also aroused at 3 h (43.9%) but showed no significant change at 6 h. *SMARCD2* was downregulated at 6 h (40.0%). *SMARCD3*, *SMARCC1* and *SMARCA4* were downregulated at 6 h after mechanical strain, with no significant difference at 3 h. *SMARCC2*, *SMARCA2* and *SMARCAB1* showed no statistical change at both 3 h and 6 h after mechanical stimuli. 

We further detected the mRNA expression (Figure 1B) and protein level (Figure 1C) of SMARCE1 in HaCaT cells at 3 h and 6 h after mechanical strain, and similar results were obtained. SMARCE1 expression was increased by 47% at 3 h and by 113% at 6 h (Figure 1D). Because of low transfection efficiency of plasmids and siRNA in osteoblast cells, HaCaT cells were used in following experiments.

### 3.2. Mechanical Stimulation Induced the Alternative Splicing of Cyclin D1

**Figure 2:**
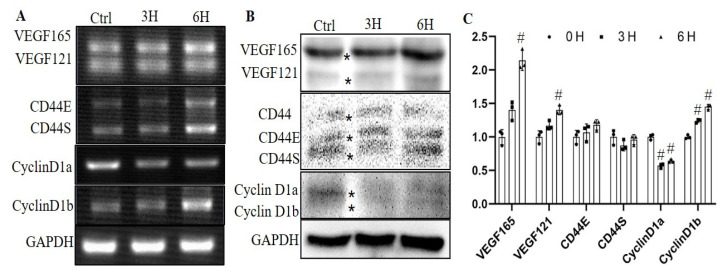
The effects of mechanical strain on the alternative splicing of VEGF, CD44 and cyclin D1. Reverse transcription semi-quantitative PCR (**A**) and Western blotting (**B**) were applied to examine the alternative splicing of *VEGF*, *CD44* and *cyclin D1* at 3 and 6 h after mechanical strain. C. The expressions of gene variants shown in B were quantified by Image J software. Data present three sets of independent experiments. #: *p* < 0.05 vs. the 0 h group. The stars * indicate the bands of gene variants.

We investigated the alternative splicing of *VEGF*, *CD44* and *Cyclin D1* in HaCaT cells after mechanical strain by RT-qPCR and Western blotting (Figure 2). RT-PCR was performed to test *VEGF165*, *VEGF121*, *CD44E*, *CD44S*, *cyclinD1a* and *cyclinD1b* at 3 h and 6 h after a cyclic stretch. Results showed that VEGF165 expression was not changed at 3 h and increased at 6 h after mechanical strain (1.3-fold), while there were no changes of VEGF121 levels at 3 h and 6 h in loaded cells. We found that cyclinD1a was downregulated at both 3 h (0.57-fold) and 6 h (0.64-fold) after mechanical strain, while cyclinD1b showed an increasing trend at 3 h (1.24-fold ) and 6 h (1.45-fold). 

### 3.3. SMARCE1 Regulated the Alternative Splicing of Cyclin D1

**Figure 3:**
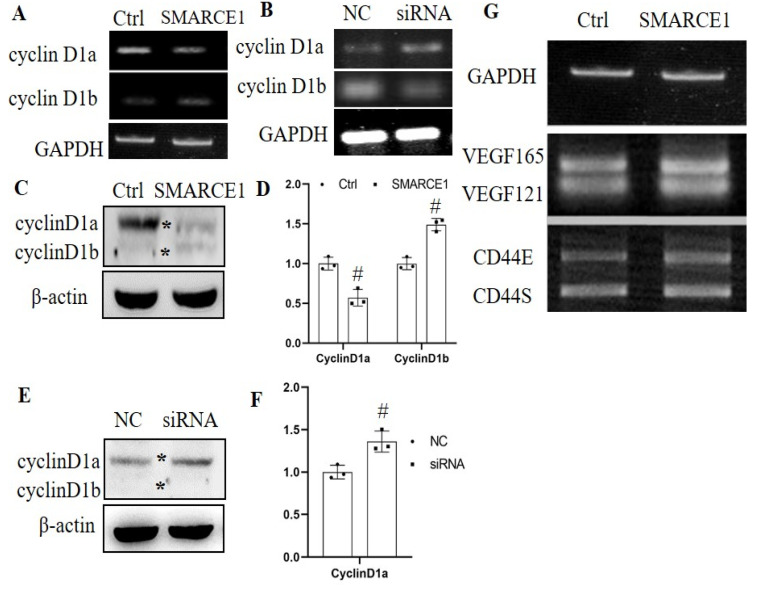
SMARCE1 influenced the alternative splicing of cyclin D1. The alternative splicing of cyclin D1 was detected by reverse transcription semi-quantitative PCR (**A**) and Western blotting (**C**) at 24 h after pcDNA3.1-SMARCE1 plasmid transfection in HaCaT cells. SMARCE1 siRNA was transfected in HaCaT cells, and the alternative splicing of cyclin D1 was examined at 24 h after transfection by reverse transcription semi-quantitative PCR (**B**) and Western blotting (**E**). Non-sense control (NC) siRNA were used as control. The expressions of cyclin D1a and cyclin D1b in C and E were quantified by Image J software and are shown in (**D**) and (**F**), respectively. (**G**). The alternative splicing of *VEGF* and *CD44* was also tested at 24 h after pcDNA3.1–SMARCE1 plasmid transfection. pcDNA3.1–GFP plasmid served as control (Ctrl) group. Data present three sets of independent experiments. # *p* < 0.05 vs. the Ctrl or NC group. The stars * indicate the bands of gene variants.

The subunit SMARCE1 of the SWI/SNF complex has been reported to be associated with regulating cell cycle-dependent transcription [20]. Therefore, we focused on the role of SMARCE1 in mechanical stress-induced *cyclin D1* alternative splicing. The results in Figure 3A showed that overexpression of SMARCE1 decreased the expression of cyclin D1a but increased cylcin D1b expression at mRNA level. Protein levels of cyclin D1a decreased by 43% and cyclin D1b increased by 49% after SMARCE1 overexpression (Figure 3C,D). Silencing of SMARCE1 by siRNA showed an opposite effect on the alternative splicing of cyclin D1 (Figure 3B,E,F). The effects of SMARCE1 on alternative splicing of VEGF and CD44 were also tested at 24 h after pcDNA3.1– SMARCE1 transient transfection into HaCaT cells. The results in Figure 3G showed that overexpression of SMARCE1 had no significant differences on the alternative splicing of *VEGF* and *CD44*. 

### 3.4. SMARCE1 Interacted with SRSF1

**Figure 4:**
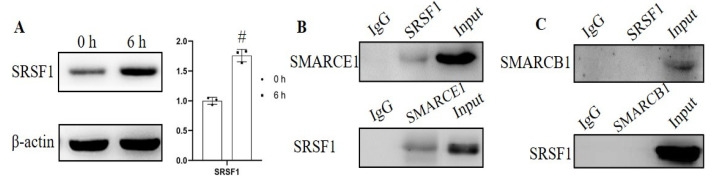
SMARCE1 interacted with SRSF1. (**A)** The SRSF1 expression was tested in HaCaT cells by Western blotting at 6 h after mechanical strain, and the bonds were quantified by Image J software. (**B**) Co-immunoprecipitation assay (Co-IP) confirmation of the interaction between SMARCE1 protein and SRSF1. (**C**) The interaction between SMARCB1 and SRSF1 was examined by Co-IP assay. Data present three sets of independent experiments. # *p* < 0.05 vs. the 0 hgroup.

**Figure 5:**
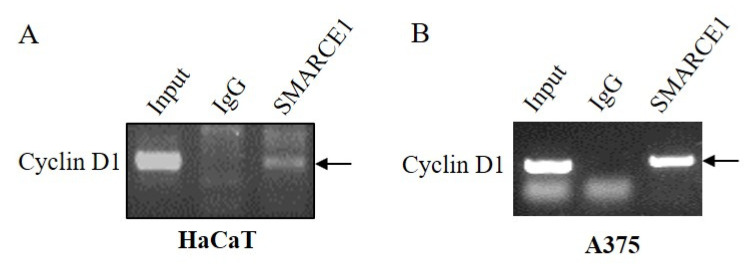
The binding between SMARCE1 and *cyclin D1* pre-mRNA. The binding of SMARCE1 to pre-mRNA of *cyclin D1* was detected by reverse transcription PCR (RIP) assay in HaCaT (**A**) and A375 (**B**) cells. Arrows indicated the cyclinD1 bond.

SR and hnRNP proteins are crucial factors directly binding to splicing elements regulating gene alternative splicing. SRSF1 (also named ASF/SF2), an important SR protein, was capability of influencing the splicing of *cyclin D1* to increase the expression of cyclin D 1b [21]. However, whether SRSF1 responds to mechanical stimuli is still unknown. Our immunoblotting results showed that mechanical stress resulted in upregulation of SRSF1 in HaCaT (1.76-fold, Figure 4A). Co-immunoprecipitation experiments from HaCaT cell extracts revealed the interaction of SMARCE1 with SRSF1 (Figure 4B). Another SWI/SNF complex subunit SMARCB1 (SNF5/INI1) was reported to repress cyclin D1 transcription [22]; therefore, we then tested to see whether SMARCB1 could also interact with SRSF1, and no association was detected between SMARCB1 and SRSF1 (Figure 4C). To further confirm the binding of SMARCE1 to *cyclinD1* pre-mRNA, RIP assay was conducted in HaCaT cells and A375 cells (Figure 5), and the results showed the interaction between SMARCE1 and cyclinD1 pre-mRNA was positive.

## 4. Discussion

Previous studies reported that SWI/SNF complex subunits could respond to UV irradiation, ROS exposure, inflammatory stimulus etc. [11,23,24]. In addition, we further test the alteration of SWI/SNF subunits after mechanical strains, and found significant changes of several subunits including SMARCE1, SMARCD1, SMARCD2, SMARCD3, SMARCC1 and SMARCA4 post-mechanical stimulus (Figure 1). The ARID1A subunit of the SWI/SNF complex also could be modulated by mechanical signals in cells according to Chang’s study [17]. Mechanical stimuli lead to various gene expression alteration in cells through mechanotransduction mechanisms by which cells integrate physicochemical signals into cellular biological events [25,26]. Physicochemical networks of protein assemblies coupling the cytoskeleton to the nucleus have now been elucidated, resulting in a prestressed nuclear organization in living cells which perhaps serve as a substrate for transducing mechanical signals to the nucleus [26,27]. Mechanical stimulus-induced changes in chromatin organization could lead to binding or differential accessibility of DNA regulatory factors that are involved in RNA splicing or gene transcription [28]. Several subunits of the SWI/SNF complex including Brm, Brg1, BAF155 and SNF5/INI1 were reported involving the gene alternative splicing process [14,15,16]. In this study, we demonstrated that the alternative splicing process of *cyclin D1* was affected by mechanical strains (Figure 2) and SMARCE1 could regulating the alternative splicing process of cyclin D1 (Figure 3). The regulator roles of other subunits responding to mechanical strains in gene splicing need further investigation. In addition, the ARID1A subunit exerted itself as an inhibitor of the pro-oncogenic transcriptional coactivators YAP and TAZ to regulate downstream related gene expression [17]. SNF5/INI1 repressed *cyclin D1* transcription leading to cell cycle arrest [22]. Brm-subunit targeting genes were identified by RNA-seq and chromatin immunoprecipitation (ChIP)-seq techniques [29]. Works from these laboratories suggest the widespread regulation roles of the SWI/SNF complex in gene transcription. Therefore, the SWI/SNF complex probably, or at least partly, mediated the mechanotransduction dependent RNA splicing or gene transcription. However, the function of SMARCE1 on gene transcription regulation is still not fully elucidated. 

The spliceosome comprises five small nuclear RNAs (snRNAs) and associated regulatory factors, catalyzing constitutive and alternative splicing [30]. Regulatory proteins including splicing factors involved in modulating splicing reaction; the splicing factors, such as SR protein, hnRNPs and etc., acted as activators or repressors of splicing by binding to exonic or intronic enhancer or silencer elements. SRSF1, a SR protein, was able to regulate the alternative splicing of the cyclin D1 gene [21], and our results showed that SMARCE1 could bind to SRSF1 through co-IP assay (Figure 4), which is probably an explanation of SMARCE1 influencing the alternative splicing process of cyclin D1. Moreover, another subunit of the SWI/SNF complex, SMARCB1, was shown to be capable of repressing *cyclin D1* transcription [22], so we also tested the interaction between SMARCB1 with SRSF1, and the result showed no interaction between the two proteins (Figure 4), indicating that SMARCB1 probably affects *cyclin D1* expression at a transcriptional level rather than at a post-transcriptional level. However, the details of the interaction between SMARCE1 and snRNA were not disclosed in this study, which will be investigated in the future.

Multiple SWI/SNF subunits have been found to be mutated at high frequency across many different tumor types, implicating a tumor suppressive role of SWI/SNF [31]. Several subunits of the SWI/SNF complex including SNF5 (SMARCB1/INI1/BAF47), ARID1A (BAF240A), SMARCA4 (BRG1), ARID1B (BAF250B), ARID2 (BAF200) and PBRM1 (BAF180) exert critical tumor suppression activities, through inhibiting oncogenic transcription, cell cycle, epigenetic instability and etc. [32]. However, other subunits such as SMARCE1 (BAF57), SMARCA2 (BRM) and SMARCAD1 (BAF60a) were implicated to promote cancers [33,34,35]. SMARCAE1 (BAF57) is elevated in a subset of tumors that, by interacting with androgen receptor, promotes prostate cancer progression [33,36]. In our study, we found that SMARCE1 (BAF57) could regulate the alternative splicing of *cyclin D1* to generate *cyclin D1b* isoform (Figure 3). We found that cyclin D1b could act as a mediator of aberrant cellular proliferation in cancer [37]. It is indicated that the effect of SMARCE1 (BAF57) on regulating cyclin D1 splicing may also contribute to cancer progression. We also examined the influences of mechanical strain and SMARCE1 overexpression on the splicing of cyclinD1 in skin melanoma A375 cells and found that mechanical stimulation and SMARCE1 both could increase the cyclinD1a and cyclinD1b expression (Supplementary Figure S1). RIP assay in A375 cells (Figure 5B) provided confirmation that SMARCE1 also could bind to cyclin D1 pre-mRNA, indicating the important role of SMARCE1 in regulating the alternative splicing of cyclin D1 gene in skin cancer cells. CCK8 assay data provided confirmation that overexpression of SMARCE1 increased the cell proliferation by 27% (Supplementary Figure S1D). We noticed that substrate stiffness can affect the migration of cancer cells and the stiffness of cancer tissues is higher than that of normal tissues [38,39], suggesting that SMARCE1 (BAF57), responding to mechanical stimulation, is probably a novel factor mediating mechanotransduction signal pathways in cancers.

## 5. Conclusions

In conclusion, SMARCE1 (BAF57) was upregulated after mechanical strain, and regulated the alternative splicing of *cyclin D1* gene to generate a *cyclin D1b* variant. SRSF1 splicing factor is probably involved the process of SMARCE1 regulating *cyclin D1* alternative splicing because of the interaction between SRSF1 and SMARCE1.

## Figures and Tables

**Table 1:** Primers.

**β-actin**	**F**	**5′CTCTGGCTCCTAGCACCATGAAGA’3**
	**R**	**5′GTAAAACGCAGCTCAGTAACAGTCCG’3**
**BAF53a/ACTL6A**	F	5′TTGATTTCCCCACGGCTATCG’3
	R	5′AGGCTGGCTTCGGATTTGAC’3
**BAF57/SMARCE1**	F	5′CCATACAGTCATCTCGCCTACA 3′
	R	5′GGAATCGTAATGCCAGAGGAC 3′
**BAF60a/SMARCD1**	F	5′CAGCAGGCGGTCCAAAATC 3′
	R	5′GCCGCTTCCTCATAATAGTCTGG 3′
**BAF60b/SMARCD2**	F	5′CCAGCGCCGAGGGTTAAAG 3′
	R	5′GCTTCCTCTCGAAAGCTAAAAGA 3′
**BAF60c/SMARCD3**	F	5′AGCTGCGCCTTTATATCTCCA 3′
	R	5′GAGTCTTCCGCATCAGGCTT 3′
**BAF155/SMARCC1**	F	5′ACACGGTGTCCCAGCTAGATT 3′
	R	5′CCACCAGTCCAGCTAGTGTTTT 3′
**BAF170/SMARCC2**	F	5′CAGAACCGCCAACCAACAAG 3′
	R	5′AGGAAACATTTGATCGGCAGT 3′
**Brg1/SMARCA4**	F	5′ TACCCCGACGAGATAGAGT 3′
	R	5′CACGTAGTGTGTGTTAAGGACC 3′
**BRM/SMARCA2**	F	5′GTCACAACGCACAGACATTCA 3′
	R	5′AGGACAATGGAGTCTTCGTAGA 3′
**SNF5INI1/SMARCB1**	F	5′GCTCCGAGGTGGGAAACTAC 3′
	R	5′CAGAGTGAGGGGTATCTCTTGT 3′
**CyclinD1a**	F	CCAGAGTGATCAAGTGTGAC
	R	CAAGGAGAATGAAGCTTTCCCTT
**CyclinD1b**	F	CCAGAGTGATCAAGTGTGAC
	R	GGGACATCACCCTCACTTAC
**VEGF**	F	GAGATGAGCTTCCTACAGCAC
	R	TCACCGCCTCGGCTTGTCACAT
**CD44**	F	GATGGAGAAAGCTCTGAGCATC
	R	TTTGCTCCACCTTCTTGACTCC

## Data Availability

Not applicable.

## Supplementary Materials

The following are available online at https://www.mdpi.com/2073-4425/12/2/306/s1, Figure S1: The mechanical strain and SMARCE1 influenced the the alternative splicing of cyclin D1 in A375 cells.
