# Supplementary figures and images for: BAF57/SMARCE1 Interacting with Splicing Factor SRSF1 Regulates Mechanical Stress-Induced Alternative Splicing of Cyclin D1
Source: Genes (Basel). 2021 Feb 21;12(2):306. doi: 10.3390/genes12020306 (PMC7927079; doi:10.3390/genes12020306)

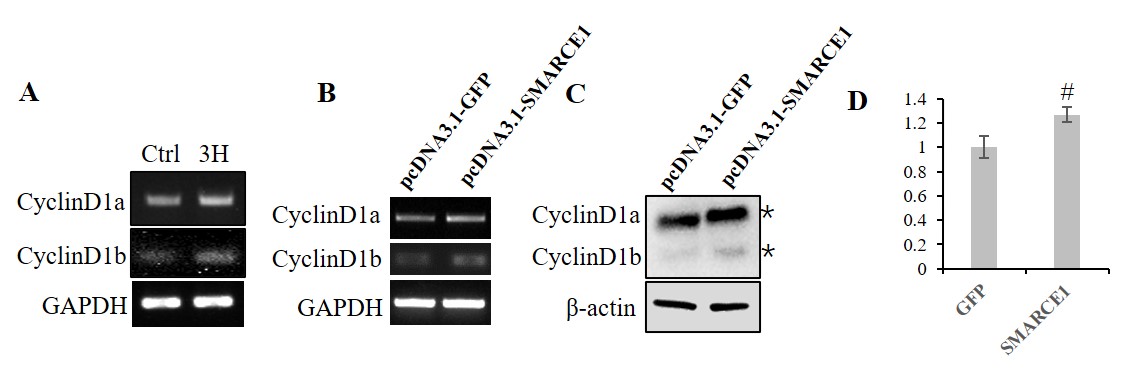

Supplement: Supplementary file 1 [file genes-12-00306-s001.zip › genes-1008861-supplementary.jpg]
